# Prevalence and impact of polypharmacy in older patients with type 2 diabetes

**DOI:** 10.1007/s40520-022-02165-1

**Published:** 2022-06-20

**Authors:** Francesca Remelli, Maria Giorgia Ceresini, Caterina Trevisan, Marianna Noale, Stefano Volpato

**Affiliations:** 1 Department of Medical Sciences, University of Ferrara, Ferrara, Italy; 2 Aging Research Center, Department of Neurobiology, Care Sciences and Society, Karolinska Institutet and Stockholm University, Stockholm, Sweden; 3 Neuroscience Institute, National Research Council, Via Giustiniani 2, 35128 Padua, Italy

**Keywords:** Diabetes, Polypharmacy, Aged, Mortality, Review

## Abstract

**Background:**

Polypharmacy is a prevalent condition in older adults, especially those with multiple chronic diseases, and has been largely associated with adverse outcomes, including disability, hospitalizations, and death.

**Aims:**

This systematic review focused on diabetes and aimed to investigate the prevalence and impact of polypharmacy in older adults affected by such disease.

**Methods:**

Observational (either cross-sectional or longitudinal) or experimental studies investigating the frequency and impact of polypharmacy in older adults with diabetes were identified from scientific databases and grey literature until August 2021. The prevalence and the 95% Confidence Interval (95% CI) of polypharmacy in older people with diabetes were summarized by a random-effects meta-analysis.

**Results:**

From a total of 1465 records, 9 were selected for the qualitative synthesis, and 8 for the quantitative synthesis. Most studies defined polypharmacy using a cut-off for the minimum number of medications ranging from 4 to 6 drugs/day. The pooled prevalence of polypharmacy in older people with diabetes was 64% (95% CI 45–80%). Considering studies that used the same definition of polypharmacy (i.e. ≥ 5 drugs/day), the pooled prevalence was 50% (95% CI 37–63%). The between-studies heterogeneity was high. Across the selected studies, polypharmacy seemed to negatively influence both diabetes-specific (poor glycemic control and risk of hypoglycemia) and health-related (risk of incident falls, syncope, hospitalization, and death) outcomes.

**Conclusion:**

This systematic review confirms the high prevalence of polypharmacy in older people with diabetes and its strong impact on several health-related outcomes, including mortality. These results strengthen the need to improve care strategies for management of these patients.

**Supplementary Information:**

The online version contains supplementary material available at 10.1007/s40520-022-02165-1.

## Introduction

Polypharmacy, defined as the concurrent and regular use of multiple medications, has been associated with unfavourable outcomes [1]. These include, but are not limited to, nonadherence to prescribed medications, drug–drug interactions, inappropriate prescriptions, and higher risk of hospitalizations and mortality [2].

Several works have shown that the frequency of polypharmacy increases with advancing age [3, 4], in parallel with the accumulation of chronic diseases [5]. In this context, the interactions between multiple drugs and diseases can lead to more challenging management and control of chronic conditions. Among the most prevalent chronic diseases in older people, diabetes mellitus can affect up to 19.3% of individuals aged 65 years or older (135.6 million people in the world) [6]. According to previous works, over 80% of individuals with diabetes suffer also from other chronic diseases [5, 7], making them more likely to present polypharmacy [8] and its detrimental consequences. These patients may, therefore, not only be exposed to the negative consequences of polypharmacy per se*,* but the presence of polypharmacy could also lead to suboptimal glycaemic control and in turn to increased risk of long-term diabetes complications, as suggested by some works [2].

Although polypharmacy is an important topic for clinical practice, studies on this topic have increased only over the last 10–15 years and the available evidence in terms of prevalence, related outcomes, and contrasting interventions, is highly heterogeneous [9]. Moreover, there are no consistent data on the extent to which polypharmacy affects specific categories of patients, such as individuals with type 2 diabetes, and how it can impact diabetes management and outcomes.

In this systematic review and meta-analysis, we aimed at summarizing the current literature on the prevalence and impact of polypharmacy in older adults with diabetes, to offer insights that may improve the clinical management of these patients.

## Methods

This work was conducted in line with the Preferred Reporting Items for Systematic reviews and Meta-Analyses statement (for the checklist, please see Appendix 1).

### Literature search

The literature search was performed in Web of Science, PubMed, and Cochrane Library datasets from inception to August 9th, 2021. To optimize the identification of eligible studies, we also examined the references of selected works and previous reviews on the topic [10, 11], and we searched in the grey literature (e.g. Ph.D. and master theses) through EBSCO Open Dissertations dataset. No restrictions by language or geographical area were applied.

### Search strategy and criteria of eligibility

This systematic review aimed at investigating the prevalence and impact of polypharmacy in older adults with diabetes. The PECOS criteria identified to address these aims were: older adults with diabetes (*population*), polypharmacy (*exposure*), no polypharmacy (*comparison*), prevalence or risk (*study design*). Since we did not focus on any specific *outcome*, we did not set any restriction on such criterion. Based on the PECOS criteria, the following key themes were, therefore, included in the search strategies: polypharmacy, older people, diabetes mellitus, and prevalence or risk (for details, please see Appendix 2).

To be eligible, studies had to include diabetic individuals with at least 60% of people aged ≥ 65 years or had to present results separately for older people with diabetes; had to report information on polypharmacy, irrespective of the number of medications used as a cut-off; had to evaluate either the prevalence of polypharmacy or the association between polypharmacy and health-related outcomes; and, had to have an observational (either cross-sectional or longitudinal) or experimental study design.

### Selection of the studies

Two researchers (MGC and FR) independently performed a first screening of the identified studies based on titles and abstracts. The records found to be eligible at this step underwent a second selection based on the review of the full-text. At each step, the researchers discussed possible disagreements in the study selection until reaching a consensus and, if needed, a third independent researcher (CT) was involved in the discussion.

### Quality assessment

Two researchers (MGC and FR) independently assessed the quality of the selected studies using the National Institutes of Health (NIH) tool for cross-sectional and cohort studies [12]. This tool includes 14 items and an overall rating that classified the quality of the studies as good, fair, or poor (for details, please see the Supplementary Table S1 footnotes).

### Data extraction

From the full-text of the included studies, two independent researchers (MGC and FR) extracted data in a structured form. For each study, the following information was obtained: first author’s last name, year of publication, study design (and follow-up time, if applicable), study population, age, sex, definition of polypharmacy, outcome, prevalence of polypharmacy (among individuals with diabetes), main results, and conclusions. One study reported estimates for different observation years [13]; in this case, the most recent estimate was prioritized. When only the median number of medications per day was reported, we derived a frequency estimate, as appropriate [14].

### Statistical analysis

The pooled prevalence and the 95% Confidence Interval (95% CI) of polypharmacy in older people with diabetes were estimated from the included studies through a random-effects meta-analysis. For this analysis, we included the studies that defined polypharmacy considering a minimum number of medications ranging from 4 to 6 drugs/day. As a sensitivity analysis, we considered only the studies that explicitly reported the prevalence of polypharmacy defined as the use of ≥ 5 drugs/day. The between-studies heterogeneity was tested using the Chi-squared test, setting a *p* value < 0.10 as significant, and expressed through the *I*-squared statistic (*I*^2^), considering a value > 75% to indicate the presence of high heterogeneity [15]. Analyses were performed using the *meta* package of R version 4.0.5 [16].

## Results

**Fig. 1:**
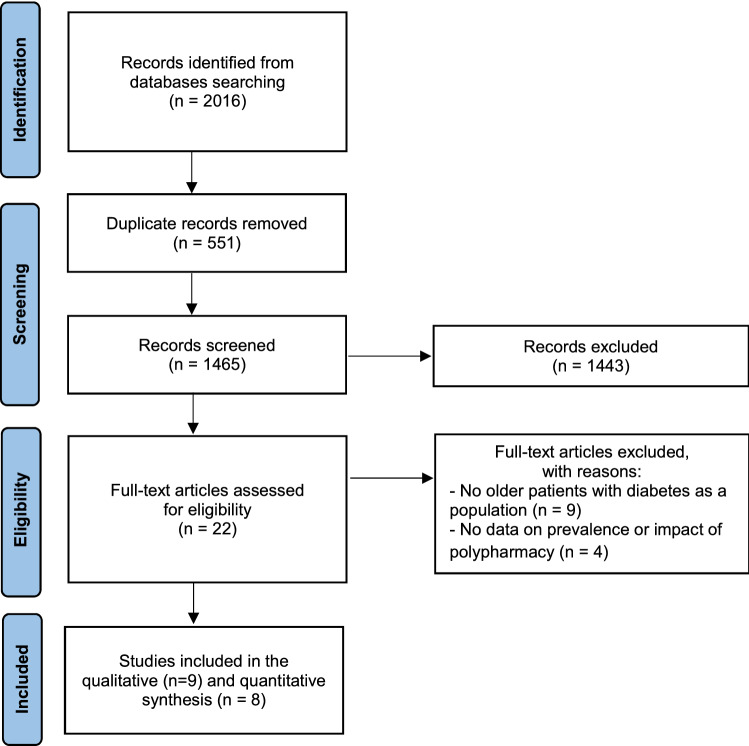
Flow diagram of the study selection

Based on the literature search, we identified 2016 records and, after the duplicate removal, 1465 underwent the title-abstract screening, finding 22 eligible studies (Fig. 1). Of these, following the full-text selection, nine records were finally included for the qualitative synthesis, and eight for the quantitative synthesis of the results.

The main characteristics of the studies included are reported in Table 1. As shown, most studies had a cross-sectional design, except for three longitudinal (two retrospective and one prospective) studies [14, 17, 18]. Concerning the study country, four works were conducted in Europe [13, 18–20], two in the United States [14, 17], one in Canada [21], one in Taiwan [22], and one in Kurdistan [23]. Almost all studies involved patients ≥ 65 years with diabetes, while Oktora et al. [13] and Yang et al. [22] involved also younger individuals (≥ 45 and > 60 years, respectively). The mean age of the enrolled samples was about 75 years, except for the work of McCracken et al. [21], performed on older patients (mean age 85 years). Concerning the definition of polypharmacy, four studies considered the usual consumption of 5 or more medications per day [13, 14, 18, 20, 22], two studies of > 5 drugs/ day [17, 19], one of ≥ 4 drugs/day [4], and one of ≥ 9 drugs per day [21]. One work reported the median number of medications taken, but not the frequency of polypharmacy [14]. The study quality evaluated through the NIH tool was good for 3 and fair for 6 records; no studies were judged as with poor quality (Supplementary Table S1).

**Table 1:** Main characteristics of the nine identified studies

Author/year	Cohort (Country)	Study design (observation period)	Population characteristics	Age (years)	Sex (F)	Definition of polypharmacy	Outcome	Prevalence of polypharmacy	Results	Conclusions
Al-Musawe et al., 2020 [19]	Population-based study, Portugal	Cross-sectional	670 individuals with diabetes	Mean 73.0 (SD: 6.2)	49.6%	> 5 drugs	Quality of Life (QoL)	72.1%	Polypharmacy was associated with severe problems in mobility (*p* < 0.001), usual activity (*p* < 0.001), personal care (*p* < 0.001), pain (*p* < 0.001), anxiety and depression (*p* = 0.037), and low QoL (OR 1.80, 95% CI 1.15–2.82)	Polypharmacy is associated with worse QoL in older adults with diabetes
Amin et al*.,* 2021 [23]	Population-based study, Kurdistan	Cross-sectional	150 individuals with diabetes ≥ 65 years	Mean 77.7 (SD: 7.11)	59%	≥ 4 drugs	Fall history	61%	Polypharmacy was highly prevalent among older adults with diabetes. No estimates on the association between polypharmacy and fall history were provided	Polypharmacy is a prevalent condition in older adults with diabetes
Bernier et al., 2012 [17]	Population-based study, United States	Retrospective (2 years)	120 individuals with diabetes ≥ 65 years	Median 75 [IQR 69–82]	57.5%	> 5 drugs	Glycemic control (HbA1c blood level)	97.5%	The total number of medications took daily was inversely associated with glycemic control (OR 0.28, 95% CI 0.10–1.80, per each 1-drug increase)	Polypharmacy is associated with poor glucose control in diabetes
Forbes et al*.,* 2016 [18]	THIN data set, United Kingdom	Prospective (10 years)	337,635 individuals (35,717 diabetic) ≥ 70 years	Diabetic: Mean 78.98 (SD 6.36) Nondiabetic: Mean 78.13 (SD 5.82)	Diabetic: 60.7% Nondiabetic: 52.3%	Four groups based on the number of drugs: 0–2 3–4 5–6 ≥ 7	1, 5, 10-year mortality	0–2 drugs: 33.5% 3–4 drugs: 35.8% 5–6 drugs: 23.4% ≥ 7 drugs: 7.3%	Polypharmacy was associated with higher mortality, with stronger results in the younger age groups (70–74 and 75–79 years). The association was confirmed both in diabetic and, more markedly, in non-diabetic individuals	Polypharmacy is associated with higher mortality both in diabetic and non-diabetic older adults
Kabue et al*.*, 2019 [14]	SUPREME- DM DataLink project, United States	Retrospective (1 year)	120,256 individuals with diabetes ≥ 65 years	Mean 73 (SD: 6.8)	49.5%	5–9 drugs (polypharmacy) ≥ 10 drugs (hyper-polypharmacy)	Hypoglycemia, hip fractures, syncope, emergency department (ED) or hospital admissions, 1-year mortality	Prevalence: N/A Number of medications, median (5) and mean 5 (SD: 3.4)	Compared with the use of < 5 drugs/day, hyper-polypharmacy was associated with an increased risk of hypoglycemia (OR 2.46; 95% CI 1.59–3.82). Both polypharmacy and hyper-polypharmacy were associated with higher odds of syncope, ED or hospital admissions, and 1-year mortality. No significant results were observed for hip fractures	The use of multiple drugs is associated with a higher risk of experiencing hypoglycemia, syncope, hospital admission, and death
McCracken et al., 2017 [21]	Population-based study, Canada	Cross-sectional	214 nursing home residents (57 with diabetes)	With polypharmacy: Mean 84 (SD: 10) Without polypharmacy: Mean 86 (SD: 9)	68.7% (total sample)	≥ 9 drugs	Glycemic control (HbA1c blood level)	57.9%	Polypharmacy showed a marginal significant association with overtreated diabetes (RR 4.0, 95%; CI 0.97–16.41; *p* = 0.05), defined as taking at least one hypoglycemic drug and having a HbA1c $\le$ 7.5%	Polypharmacy is associated with more intensive treatment of diabetes
Noale et al*.,* 2015 [20]	METABOLIC Study data set, Italy	Cross-sectional	1,342 individuals with diabetes ≥ 65 years	Mean 73.3 (SD: 5.5)	47.5%	≥ 5 drugs	Hypoglycemia, chronic diabetes-related complications	57.1%	Patients with polypharmacy had a longer median duration of diabetes (10 vs 8 years, *p* < 0.001), higher BMI (29.4 ± 5.0 kg/m^2^ vs 28.5 ± 4.7 kg/m^2^, *p* = 0.001) and prevalence of diabetes complications including coronary and cerebrovascular diseases, peripheral neuropathy, nephropathy, and retinopathy. Hypoglycemic events in the previous 3 months were more frequent in polypharmacy group (15.8% vs 6.9%, *p* < 0.001)	Older diabetic patients with polypharmacy have more hypoglycemic events and diabetes-related complications compared to those using fewer drugs
Oktora et al*.,* 2021 [13]	IADB.nl database, Netherlands	Cross-sectional (over 5 years)	24,809 individuals with diabetes ≥ 45 years (15,267 ≥ 65 years) in 2016	61.5% of the total sample with age ≥ 65 years	N/A	≥ 5 drugs	Potentially inappropriate medication (PIM) prevalence	66.2% (in 2016)*	From 2012 to 2016 there was a slight increase in the prevalence of polypharmacy. Instead, the frequency of older people with polypharmacy and at least one PIM showed a decreasing trend from 2012, and it was 24.9% in 2016	Almost a quarter of older diabetic patients with polypharmacy had also at least one PIM
Yang et al*.,* 2019 [22]	Population-based study, Taiwan	Cross-sectional	316 individuals with diabetes > 60 years	Mean 69.6 (SD: 6.6)	49%	≥ 5 drugs	Quality of Life (QoL)	46.6%	Polypharmacy was the most common geriatric syndrome in individuals with diabetes. There were no differences in QoL scores between individuals with vs without polypharmacy. The number of medications significantly contributed to the social domain of QoL, and marginal significant results were found for the physical and environmental domains	Polypharmacy is highly prevalent in older adults with diabetes. The use of multiple medications may be negatively associated with QoL

*The prevalence of polypharmacy refers to individuals aged 65 years or older

Of the nine included studies, four evaluated polypharmacy in respect to diabetes-related outcomes, such as glycaemic control (defined optimal with HbA1c blood level < 7% [17] or < 7.5% [21]), hypoglycaemic events [14, 20], and complications of the disease [20]. Other health-related outcomes included mortality [14, 18], and incident falls, hip fractures, syncope, and Emergency Department (ED) or hospital admissions [14, 23]. Two studies investigated the association between polypharmacy and Quality of Life (QoL), assessed by the EuroQoL five-dimensional questionnaire [19] and the WHOQoL-BREF Taiwan version [22]. Lastly, the prevalence of Potential Inappropriate Medication (PIM) was investigated only by one study [13].

**Fig. 2:**
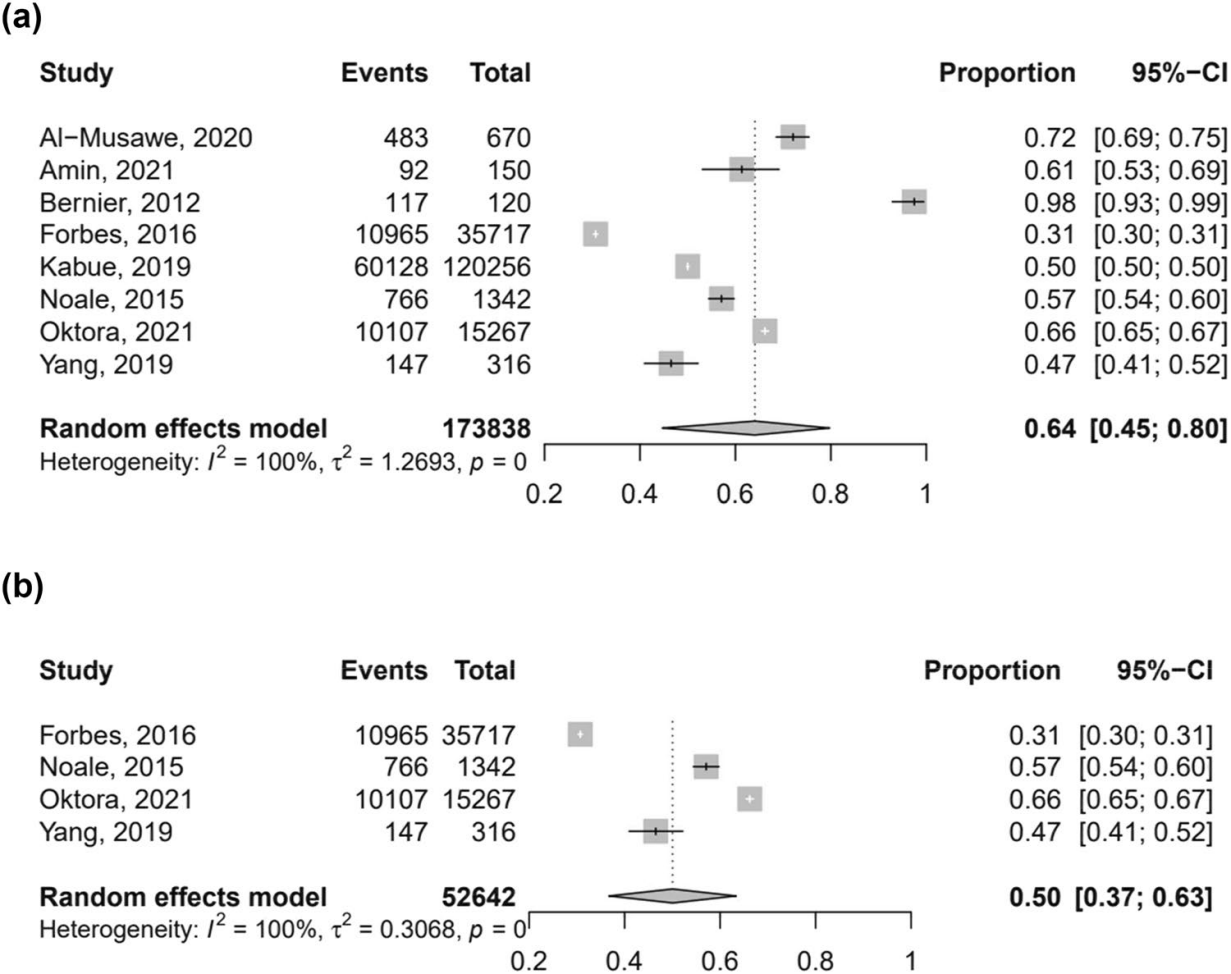
Pooled prevalence of polypharmacy in the nine selected studies (**a**) and in the four studies defining polypharmacy as ≥ 5 medications/day (**b**)

For the quantitative synthesis, we excluded the study [21] that evaluated the frequency of people using ≥ 9 drugs, which substantially differed from the cut-offs considered in the other works. Across these selected studies, the prevalence of polypharmacy ranged from a minimum of 47% [22] to a maximum of 97.5% [17]. The random-effect meta-analysis including 173,838 participants (Fig. 2a) showed that the pooled prevalence of polypharmacy was 64% (95% CI 45–80%). Through the sensitivity analysis on the four studies that used the same definition of polypharmacy (i.e. ≥ 5 drugs/day) [13, 14, 18, 20, 22], including 52,642 individuals, the pooled prevalence was 50% (95% CI 37–63%) (Fig. 2b). In both analyses, the between-studies heterogeneity was high.

The high heterogeneity that characterized the outcomes of the included studies did not allow us to perform further quantitative synthesis of the results. Considering diabetes-related outcomes, Bernier et al. reported that the total number of drugs taken daily was associated with poor glycaemic control, up to a real overtreatment of diabetes which, in that study, was defined as the prescription of at least one antidiabetic medication in individuals with HbA1c < 7.5% [17]. Both a retrospective [14] and a cross-sectional study [20] showed that polypharmacy in diabetic older people was associated with a risk of hypoglycemic events twice as higher as that of patients taking < 5 drugs/day. Kabue et al. [14] observed that polypharmacy (i.e. the use of 5–9 drugs/day) and hyper-polypharmacy (i.e. ≥ 10 drugs/day) over 1 year were associated with a higher risk of all the investigated negative outcomes (except for hip fracture), including syncope, ED or hospital admissions, and death. An association between polypharmacy and 1-, 5-, 10-year mortality was also observed in a cohort study on 337,635 individuals ≥ 70 years [18]: in the diabetic group, the risk of mortality of people taking $\ge$ 7 drugs/day was 34% higher than people without polypharmacy. Lastly, the two cross-sectional studies that considered QoL assessed through different scales, showed that the presence of polypharmacy in older people with diabetes tended to be associated with worse QoL [19, 22].

**Fig. 3:**
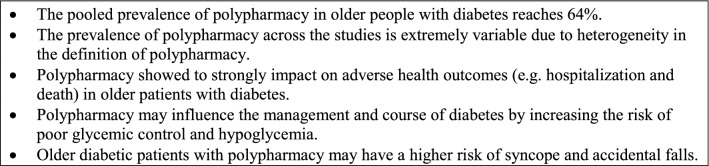
New findings highlighted by the present review

Figure 3 summarizes new findings highlighted by the present review.

## Discussion

Our study confirms that polypharmacy is a prevalent condition among older people with diabetes, characterizing almost two-third of such population. Moreover, in line with the existing literature on polypharmacy [24], we found that older individuals with diabetes who use multiple drugs may have a higher risk of several negative health-related outcomes, including poor glycaemic control [17, 21] and hypoglycaemic events [14, 20], syncope [14], poor QoL [19, 22], need for hospital-based care [14], and death [14, 18].

Across the selected studies, the pooled prevalence of polypharmacy in older people with diabetes was 64%. This picture seems to be higher compared with previous estimates considering the general population aged 65 years or older (18%) or patients affected by other diseases, such as heart disease (43%) [25]. This may be partly explained by the fact that older adults with diabetes have often multiple coexisting comorbidities such as cardiovascular, metabolic, renal, respiratory, and musculoskeletal diseases, which might require the use of multiple medications and therefore increase the chances of presenting polypharmacy [5]. In this regard, it should be mentioned that the selected studies used different definitions of polypharmacy, with the most common cut-off being the use of ≥ 5 medications/day, and only a few studies distinguishing the use of ≥ 10 medications/day [14, 21].

This systematic review suggests the presence of a strong association between polypharmacy and adverse health outcomes also in older patients with diabetes. In particular, polypharmacy showed a potential impact on the management and course of diabetes, as well as in other health domains. Concerning the first aspect, studies generally reported a higher frequency of poor glycaemic control [17, 21] and hypoglycaemic events [14, 20] among older individuals with diabetes and polypharmacy. This effect could be partly associated with the overuse of antidiabetic drugs, which is, in older and frail patients, often linked to the establishment of unrealistic glycaemic targets and can lead to unbalances between the benefits of medications for diabetes control and. the risk of hypoglycaemic events [26]. A further mechanism through which polypharmacy may increase the risk of poor glycaemic control concerns the interactions between antidiabetic drugs and other medications, which may substantially affect the pharmacokinetics of the former [13, 27]. Such drug–drug interactions, as well as the occurrence of possible adverse drug reactions, can be misinterpreted as indicators of a new disease or poor diabetes control, determining the prescription of new drugs in a process known as “prescription cascade” [28, 29].

Concerning other health-related outcomes, older diabetic patients with polypharmacy seem to be more exposed to the risk of syncope and accidental falls [14, 23]. In this regard, it is well known that the effects of autonomic neuropathy in diabetes could be exacerbated by the use of some medications, such as antihypertensive, alpha-blockers, and benzodiazepines, which may further increase the risk of falls [30–32]. Moreover, as described above, patients with polypharmacy are more likely to experience hypoglycaemic events that could represent an additional factor predisposing to falls and syncope [14, 20, 23, 33–35]. Overall, the negative impact of polypharmacy on multiple health domains in older patients with diabetes can also lead to a higher risk of hospital admissions and death [14, 18], as well as a poorer QoL [19, 22]. These effects may be mediated by the greater exposure to the complications of diabetes and use of multiple drugs, which determine a steeper loss in self-sufficiency and a greater need for medical care and assistance [26, 36–38].

The high prevalence of polypharmacy in older adults with diabetes and its associated adverse outcomes represent challenges for the clinical management of these patients. Therefore, suitable strategies to overcome this problem are necessary (Table 2). These include, for instance, regular medication review, revision of glycemic targets, and possible deprescribing tailored on each patient based on the comprehensive geriatric assessment [21, 39]. Of note, concerning the association between polypharmacy and poor QoL, deprescription may be not always the most effective solution but, together with pharmacological review, should be carefully evaluated in respect to specific patient’s health domains. As reported by the American Diabetes Association (ADA) “Standards of Medical Care in Diabetes – 2021” [26], a comprehensive assessment should be performed also to evaluate the patient’s social context and ability to self-manage the prescribed antidiabetic therapy. This is a crucial point since antidiabetic therapy often requires adjusting insulin doses based on monitoring blood glucose and this task may be especially challenging for patients with multiple chronic conditions, such as cognitive decline or visual impairment, which are frequent complications of diabetes in advanced age [39–41]. Moreover, given the importance to propose a treatment tailored on older patients with diabetes and polypharmacy, the deintensification of antidiabetic therapy using long-acting medications and the simplification of the diabetes management through practical, pharmacological or dietary strategies should be always considered [26, 42]. Evidence on the effectiveness of the above-mentioned strategies from interventional studies is still scarce [43, 44]. In particular, in the randomized controlled trial of Xu et al., a collaborative care model showed to improve the achievement of the glycemic targets and reduce the related medical costs, in diabetic patients with polypharmacy [43]. Similarly, in another trial on individuals with multimorbidity including diabetes, an interprofessional team-based approach seemed to lead to better diabetes and blood pressure control [44]. These promising results support the need for further interventional studies that could delineate the most effective and feasible strategies to manage polypharmacy in older people with diabetes.

**Table 2:** Major strategies for the clinical management of polypharmacy in older patients with diabetes

Main strategies to manage polypharmacy in older people with diabetes
Regular pharmacological review of the ongoing therapies
Pharmacological deprescription of potentially inappropriate medications
Revision of glycemic targets according to patient’s health status
Prescription of antidiabetic therapy based on patient’s social context and skills, assessed by the comprehensive geriatric assessment
Deintensification of antidiabetic therapy using long-acting medications
Promotion of healthy dietary habits (e.g. reducing the carbohydrates per meal)
Promotion of physical activity with realistic goals (e.g. walking at home at least for 15–20 min daily)

Our systematic review has some limitations that need to be discussed. As previously mentioned, the high heterogeneity of the study samples and the definitions of polypharmacy made it difficult to compare the selected records. Moreover, each health-related outcome was evaluated by not more than two studies and was generally assessed with different methods that did not allow us to perform further meta-analyses. An additional limitation lies in the lack of information on the type of medications prescribed, which could give some relevant insights into the patterns of polypharmacy and may partly explain the impact of such condition on the outcomes considered. With regards to the strengths of our work, we followed the PRISMA guidelines for reporting systematic reviews [45]. Moreover, the selection of the studies was carried out also evaluating the grey literature so that current evidence in the topic should have been extensively captured. It is worth underling also that this is a very actual issue in geriatric medicine, although nowadays is still poorly investigated.

## Conclusions

In conclusion, our systematic review highlights the high prevalence of polypharmacy in older people with diabetes and suggests that such condition may have a substantial impact on several health-related outcomes. However, future investigations using a consistent definition of polypharmacy and considering different settings (e.g. nursing home) are needed to increase evidence on this intriguing issue, with the goal of improving clinical management of such patients.

## Supplementary Information

Below is the link to the electronic supplementary material.

Supplementary file1 (DOC 99 kb)

## Data Availability

From authors upon appropriate request.
